# Over-Expression of βII-Tubulin and Especially Its Localization in Cell Nuclei Correlates with Poorer Outcomes in Colorectal Cancer

**DOI:** 10.3390/cells8010025

**Published:** 2019-01-07

**Authors:** Kseniya Ruksha, Artur Mezheyeuski, Alexander Nerovnya, Tatyana Bich, Gennady Tur, Julia Gorgun, Richard Luduena, Anna Portyanko

**Affiliations:** 1 N.N. Alexandrov National Cancer Centre of Belarus, 223040 Minsk, Belarus; Kseniyaruksha@yandex.ru; 2 Department of Pathology, Belarusian State Medical University, 220116 Minsk, Belarus; artur.mezh@gmail.com (A.M.); nam64@tut.by (A.N.); itati79@gmail.com (T.B.); a_port@mail.ru (A.P.); 3 Minsk City Clinical Oncologic Dispensary, 220013 Minsk, Belarus; getur@tut.by; 4 Department of Gastroenterology and Nutrition, Belarusian Medical Academy of Post-Graduate Education, 220013 Minsk, Belarus; julia.gorgun@mail.ru; 5 Department of Biochemistry and Structural Biology, University of Texas Health San Antonio, San Antonio, TX 78229, USA

**Keywords:** βII-tubulin, microtubules, nuclear tubulin, colorectal cancer, cancer prognosis

## Abstract

Tubulin is a heterodimer of α and β subunits, both existing as isotypes differing in amino acid sequence encoded by different genes. Specific isotypes of tubulin have associations with cancer that are not well understood. Previous studies found that βII-tubulin is expressed in a number of transformed cells and that this isotype is found in cell nuclei in non-microtubule form. The association of βII expression and its nuclear localization with cancer progression has not previously been addressed. We here used a monoclonal antibody to βII to examine patients with colorectal cancer and found that patients whose tumors over-express βII have a greatly decreased life expectancy which is even shorter in those patients with nuclear βII. Our results suggest that βII-tubulin may facilitate cancer growth and metastasis and, to accomplish this, may not need to be in microtubule form. Furthermore, βII expression and localization could be a useful prognostic marker. We also found that βII appears in the nuclei of otherwise normal cells adjacent to the tumor. It is possible therefore that cancer cells expressing βII influence nearby cells to do the same and to localize βII in their nuclei by an as yet uncharacterized regulatory pathway.

## 1. Introduction

Colorectal cancer (CRC) is the third most common cancer in men and the second in women in the United States [1]. According to the World Health Organization statistics, CRC is the fourth most common cause of death from cancer after female breast cancer, prostate cancer in men, and lung cancer, with more than 1.4 million expected cases of incidence every year [2]. Expected morbidity and mortality in 2035 are 2.4 and 1.3 million new cases, respectively [3]. Tubulin, the subunit protein of microtubules [4,5], is an α/β heterodimer [6]. Both α and β exist as isotypes differing in amino acid sequence and encoded by different genes [7,8]. Considerable evidence has accumulated that tubulin can exist in cells in non-microtubule forms [9,10,11,12,13,14]. This is especially the case for the βII isotype, which often occurs in cell nuclei, possibly in the form of a reticulum, but not as a microtubule [9]. Specific nuclear localization of βII-tubulin was demonstrated not only by immunohistochemistry using a monoclonal antibody to βII [15], but also by immunoblotting of a purified nuclear fraction, and by the fact that fluorescently labeled αβII-tubulin when micro-injected into these cells, went into the nuclei whereas fluorescently labeled, micro-injected αβIII and αβIV did not [9]. Nuclear βII is particularly common in cancer cells, less common in cultured cells, and much less common in normal cells in situ [16,17,18]. βII is commonly over-expressed in tumors where the normal cells express little or no βII [18]. This is especially significant because βII is also the tubulin isotype with which some of the most successful anti-tumor drugs, namely, paclitaxel and vinblastine, interact the best [19,20].

We previously showed that βII is expressed in cells excised from the tumors of patients with CRC, both metastatic and non-metastatic [18]. Normal colon expresses little or no βII [21,22]. We also showed that βII occurs in the nuclei of cells excised from the tumors of patients with CRC, as well as many other cancers [18]. Although the previous study only examined samples from 15 patients with CRC, no difference was observed in βII localization in metastatic and non-metastatic disease [18]. However, in some other cancers, it appeared that nuclear βII was most likely to occur in metastatic tumors [18].

In this study, we used a monoclonal antibody to βII-tubulin [15] to examine surgical material from patients with CRC and measured the life expectancies of the patients. We found that over-expression of βII was correlated with a shorter life expectancy of patients with CRC. The life expectancy was even shorter for patients in whose tumors βII was localized to the cell nuclei. We also found that otherwise normal cells close to the tumor also expressed βII and localized it to their nuclei.

Our results have both cell biological and clinical implications. They suggest that there exists an as yet uncharacterized pathway whereby βII is synthesized and localized to nuclei in both cancer cells and in nearby normal cells, somehow influenced by the cancer, and that this pathway may be correlated with increased aggressiveness of the cancer. Our findings also raise questions about the role of βII tubulin in both normal and cancerous cells. From a clinical perspective, these results also have implications about the possible prognostic utility for patients with CRC of βII expression and nuclear localization. Furthermore, the current understanding of how anti-tubulin drugs operate in cancer is that they freeze microtubule dynamics [23,24,25]. If tubulin, in non-microtubule form, is affecting cancer progression then that understanding needs to be expanded. In other words, the fact that βII apparently exists in advanced CRC in non-microtubule form raises the possibility that tubulin does not need to be in a microtubule to promote cancer cell growth and proliferation and that non-microtubule tubulin may constitute a novel and hitherto unexplored target for cancer chemotherapy, and may even have a function in normal cells.

## 2. Materials and Methods

### 2.1. Source of Patients

Investigations were carried out following the rules of the Declaration of Helsinki of 1975, revised in 2013. The research was approved by the Ethics Committee of the Belarusian State Medical University prior to commencing the study. The study included 124 patients (55 male, 69 female, median age 65.0 years old, q_1_–q_3_ 57.0–73.0 years old) with CRC (See Table S1 in Supplementary Material). All patients had had a bowel resection performed by the same surgeon in Minsk City Clinical Oncological Dispensary in 2009–2011. Patients’ follow up was carried out according to the national protocols. Progression was defined as tumor growth after radical resection of the bowel segment. The median time of dynamic follow-up was 3.56 years (q_1_–q_2_ 1.2–4.4 years, maximum 5.5 years). Medical examinations were conducted once every six months during the first two years after operation and once a year after two years after operation. Only patients who signed the informed consent were included. Results were not available to clinicians at the time of patients’ treatment and follow-up.

### 2.2. Tissue Samples

The tissue specimens were dissected from the edge of the tumors. Pathological analysis was performed on resected specimens and staged according to the American Joint Committee on Cancer [26]. For the purpose of the research the blocks containing the deepest invasive margin were selected. Non-tumor colonic mucosa or mucosa at a distance of no less than 1 cm from the tumor were used as a control group (60 cases). Features of the tumors are presented in Table S2 in the Supplementary Material.

### 2.3. Immunohistochemistry

Paraffin-embedded tissues (4-μm-thick) were cut from tissue blocks and mounted on slides coated with 3-aminopropylenetriethoxy-silane, then deparaffinized in xylene and rehydrated in alcohol. Endogenous peroxidase activity was inhibited by 3% hydrogen peroxide for 20 min. After washing twice in 0.05 M Tris buffered saline (TBS, Sigma Aldrich, Darmstadt, Germany) non-specific binding was blocked using 1% bovine serum albumin in TBS for 30 min. Antigen retrieval was carried out in a Pascal Pressure Cooker (Dako, Carpinteria, CA, USA) at 125 °C and 25 psi for 30 s with 0.01 M citrate buffer (pH 6.0). At the next step slides were incubated with primary anti-mouse monoclonal antibodies to βII-tubulin (clone JDR3B8, IgG_2b_-isotype, 1:40, BioGenex, Fremont, CA, USA) at 4 °C overnight. This antibody does not distinguish between the different forms of βII-tubulin (βIIA, βIIB and βIIC). Subsequently they were washed twice with TBS and then staining was detected by Super Sensitive^TM^ Polymer-HRP IHC Detection System (BioGenex, Fremont, CA, USA). Diaminobenzidine (DAB, DAKO, Glostrup, Denmark) was used as a chromogen. Then slides were counter-stained with hematoxylin and mounted in Cytoseal (ThermoScientific, Waltham, MA, USA). Nerve fibers and nerve ganglia were used as a positive inner control due to their intense reactivity.

### 2.4. Immunohistochemistry Evaluation

Tissue samples were analyzed using a Leica DM5000 B microscope at ×200 magnification. βII-tubulin expression was evaluated separately in the central regions of the tumor and in the deepest invasive margin. The invasive front was defined as the deepest invasive margin of the tumor within one field of vision (×200). Moreover, expression was assessed both in the nuclei and in the cytoplasm. The nerve trunks served as an internal positive control. Positive immunoperoxidase staining of nerves for βII has been previously reported for a variety of tissues [18,21]. Slides stained by immunohistochemistry without using primary antibody served as a negative control. Immunohistochemical staining was interpreted as either positive or negative and all cases were classified in groups according to the absence or presence of βII-tubulin expression. Those cases in which any number of cells showed positive nuclear or cytoplasmic staining were classified as positive while those cases with absence of brown staining were classified as negative. Most of the cases (76%) were negative for βII-tubulin. We did not observe any signs of non-specific staining as a “side effect”, for example. Usually positive staining was clearly localized to the cytoplasm or nucleus or both. During acquisition of the images the pathologist was blinded to any clinical or staging data. We previously used the same immunohistochmical approach to examine the distribution of βIII-tubulin in CRC [27].

### 2.5. Statistical Analysis

Image analysis was performed using RStudio, v. 0.98.1103 (RStudio, Inc., Boston, MA, USA). Groups were compared using the Wilcoxon test (*p*_w_). Survival curves for different groups were obtained using the Kaplan-Meier estimator and then compared by a log-rank test (*p*_lr_). Null hypothesis was rejected at *р* < 0.05.

### 2.6. Data Sharing

All the figures, original data, and protocols are available, although patient identifying information is not. Please contact Dr. Portyanko for such information.

## 3. Results

**Figure 1:**
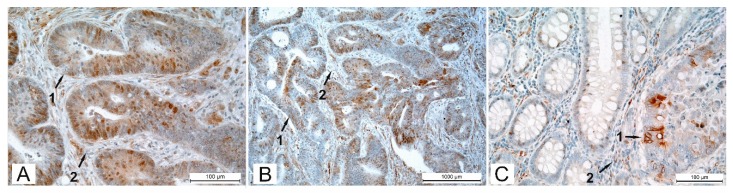
Immunohistochemical staining of βII-tubulin in colorectal cancer specimen detected by peroxidase-mediated diaminobenzidine (DAB)-staining (brown) with arrows indicating epithelial compartments of colorectal cancer (CRC) (1) and stroma (2). (**A**) Epithelial compartment of tumor cells showing moderate cytoplasmic and more intense nuclear βII-tubulin staining. There are single positive stromal cells (original magnification ×200). (**B**) Adenocarcinoma at lower magnification (original magnification ×100). There are single positive stromal cells. (**C**) Normal colonic mucosa (left) adjacent to tumor complexes (right) showing appearance of positive nuclear βII staining (original magnification ×200).

**Figure 2:**
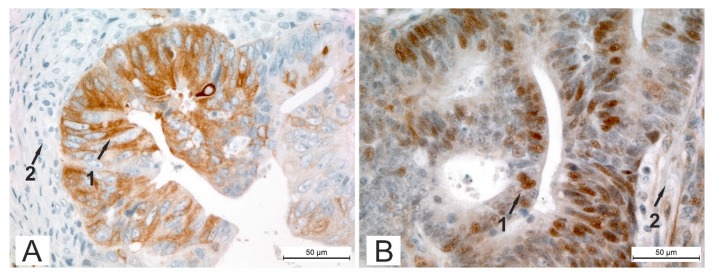
Immunohistochemical staining of βII-tubulin in CRC specimen detected by peroxidase-mediated DAB-staining (brown) with arrows indicating epithelial compartment of CRC (1) and stroma (2). (**A**) Epithelial compartment of tumor cells showing moderate cytoplasmic βII-staining, (original magnification ×400). (**B**) Epithelial compartment of tumor cells showing strong focal nuclear βII-staining, (original magnification ×400).

βII-tubulin expression was detected in 30 cases of CRC (28.0%). Cell cytoplasm showed positive staining in all the tumors (30 cases—28.0%). Furthermore, in 14 of these 30 cases βII-tubulin was present in the nuclei (14 cases—11.2%). (Figure 1A). To highlight the difference in cytoplasmic staining and nuclear staining for βII-tubulin, the samples are shown at higher magnification in Figure 2 to illustrate both cytoplasmic (Figure 2A) and nuclear (Figure 2B) staining of βII-tubulin.

In most cases fewer than 5% of the tumor cells demonstrated unambiguous expression of βII-tubulin; the intensity of this staining was variable but its presence was unambiguous. Positively stained cancer cells were either concentrated as small βII-positive foci or were diffusely scattered across the tumor (Figure 1B). No mitotic spindles containing βII-tubulin were observed. The averaged number of mitoses for every case varied from 0 to 11.3. The number of mitoses in the groups with or without βII-tubulin expression did not show a statistically significant difference either in the center (*p* = 0.64) or in the invasive front (*p* = 0.85). It was not clear if βII was forming microtubules. Both the area of the tumor center (30 tumors—24.0%) and the area of the tumor invasive front (19 tumors—15.2%) exhibited positively stained regions. (Table 1).

**Figure 3:**
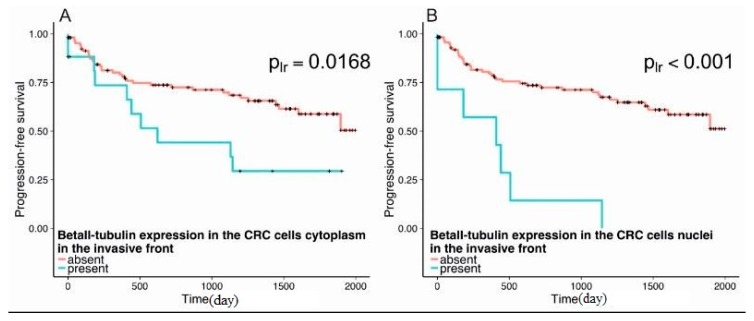
Progression-free survival in patients as a function of the presence of βII-positive staining in the invasive front. (**A**) The progression-free survival is decreased in patients with the presence of cytoplasmic βII-tubulin in the invasive front (*p*_lr_ = 0.0168). (**B**) Patients with the presence of nuclear βII tubulin staining in the invasive front demonstrate worse prognosis in comparison with patients without positive staining in the nuclei (*p*_lr_ < 0.001).

Malignant cells expressed βII-tubulin exclusively in the tumor center in 56.7% (17/30 tumors) of all cases while in 20.0% (6/30 tumors) expression was detected only in the invasive front. In the other cases both the center and the invasive front showed positive staining. The Wilcoxon matched pairs test did not reveal a statistically significant difference between the expression of βII-tubulin in the center and in the invasive front (*p*_w_ = 0.1075). It should be noted that the expression of this isotype in the invasive front was associated with higher probability of disease progression. The results showed that the presence of βII was associated with decreased survival, a pattern that was even more striking when βII was in the nuclei. This difference was revealed both for cytoplasmic (*p* = 0.0168) and nuclear (*p* = 0.0000) patterns of immunostaining (Figure 3). The log rank test showed that there was no difference in survival time between the groups with and without the expression of βII-tubulin either in the cytoplasm (*p* = 0.452) or in the nuclei (*p* = 0.245) of cancer cells in the tumor center.

One of the observations in our study was that 24 of 58 tumors containing adjacent normal mucosa also had positive nuclear βII-staining in this otherwise normal area. However, the resection margins, which are parts of apparently non-tumorous bowel mucosa after surgical resection of bowel segment with a tumor, did not reveal positive nuclear or cytoplasmic βII-staining (Figure 1C).

## 4. Discussion

The findings reported here raise questions of both clinical and biological significance. The data clearly show that over-expression of βII in CRC is associated with shortened survival and that this is even more pronounced for patients with nuclear βII. It is interesting that the shapes of the two curves are different (Figure 3). Survival for the first 400 days is not very different between βII over-expression and localization of βII in the nucleus, but after 400 days, survival drops off very steeply for the latter group, to the point where very few survive after 1200 days. Granted that there are other mechanisms for prognosticating survival in patients with CRC [28,29], then, at the very least, observation of βII may provide, if the test is further developed, another predictive tool, one requiring only a biopsy. Furthermore, it is conceivable that the presence of nuclear βII may indicate somewhat poorer survival overall with even poorer survival after 500 days; this kind of high-resolution prognostication may be useful for patients.

At this point, it is worth mentioning that some studies have found that mRNA levels for the two forms of βII-tubulin (βIIa and βIIb) are very low in certain colon cancer cell lines [30] and in some cases of CRC [31,32]. However, there is also evidence for over-expression of βII-tubulin in other cases of CRC and other cancers, sometimes associated with increased drug resistance [33,34,35]. In either case, we must recall that mRNA expression and protein expression are not always correlated and that, if we extrapolate from studies suggesting that expression of βII mRNA may be low in the cancers we have studied, this putative discrepancy raises the possibility that the issue in these tumors is one of decreased degradation of βII. Future studies may resolve this issue.

It is not yet clear what functions βII and nuclear βII serve for cancer cells. Some evidence in the literature raises the possibility that βII-tubulin may be involved in membrane rearrangements [11,36,37], some of which may involve microtubules [38]. Since cancer cells grow, divide, and migrate, it is reasonable to speculate that new membrane is often being made or rearranged [39] and hence that βII could be useful in this regard, perhaps mediating microtubule-membrane connections. This could account for the over-expression of βII that has been observed in CRC and a large number of other cancers [18]. The function of nuclear βII in cancer cells, however, remains a mystery. There is evidence, however, that nuclear βII can interact with antitumor drugs, such as paclitaxel and vinblastine [17,40,41], indicating that, if nuclear βII is indeed present in CRC and other tumors, then chemotherapeutic strategies may need to take this into account.

The observation that otherwise normal cells adjacent to the tumor express βII, including nuclear βII (Figure 1C), has been made before for a variety of tumors [18], but not in any quantitative manner, as we have done here. There are three implications of this finding. First, if a tumor is searched for in a biopsy, then if the probe misses the actual tumor, observation of cells containing βII and, even more strikingly, nuclear βII, would imply the nearby presence of a tumor, which could powerfully augment the utility of a biopsy. Second, the mechanism by which βII is made in otherwise normal cells adjacent to the tumor and localized to the nuclei is unknown. Our observations imply the existence of a hitherto unknown signaling pathway that affects tubulin biosynthesis and subcellular localization. Perhaps the pathway involves production of a substance by the tumor that influences the nearby cells to behave in this way. Third, the mechanism by which such a substance might enter the normal cells is also unknown. It may require a nanotubule of some kind. Further exploration of these mechanisms could not only add to our knowledge of basic cellular regulatory pathways, but also reveal hitherto unsuspected targets for novel chemotherapies.

## Figures and Tables

**Table 1:** βII-tubulin expression in colorectal cancer (CRC).

Intensity of Staining	βII-Tubulin Expression					
	Tumor Center		Invasive Front		Normal Mucosa	
	N	%	N	%	N	%
Negative	95	76.0	106	84.8	34	58.6
Positive	30	24.0	19	15.2	24	41.4
All	125	100	125	100	58	100

## Supplementary Materials

The following supplementary materials are available online.
