# Supplementary material for: Over-Expression of βII-Tubulin and Especially Its Localization in Cell Nuclei Correlates with Poorer Outcomes in Colorectal Cancer
Source: Cells. 2019 Jan 7;8(1):25. doi: 10.3390/cells8010025 (PMC6357106; doi:10.3390/cells8010025)
Supplement: Supplementary file 1 [file cells-08-00025-s001.pdf]

Table S1. Clinical data about the patients

**Feature**	**n**	**%**
Age at the moment of the operation		
≥ 70	46	37.1
< 70	78	62.9
All	*124*	*100.0*
Gender		
Female	69	55.6
Male	55	44.4
All	*124*	*100.0*
Synchronous distance metastases		
No	103	83.1
Yes	21	16.9
All	*124*	*100.0*
Clinical stage		
I	14	11.3
II	41	33.1
III	50	40.3
IV	19	15.3
All	*124*	*100.0*
Neoadjuvant radiotherapy of cancers of the rectum		
No	20	60.6
Yes	13	39.4
All	*33*	*100.0*
Adjuvant 5-FU chemotherapy in patients with clinical stage II-IV		
No	59	53.6
Yes	51	46.4
All	*110*	*100.0*
Progression after 3,56 years (q_1-q_3 1,24 – 4,36)		
No progression	78	62.9
Metachronous metastases	36	29.1
Peritoneal carcinomatosis	4	3.2
Regional recurrence	6	4.8
All	*124*	*100.0*
Outcome after 3,56 (q_1-q_3 1,24 – 4,36)		
Alive	81	65.3
Dead	43	34.7
All	*124*	*100.0*

Table S2. Features of the tumors

Feature		n	%
Bowel segment			
	cecum	18	14.4
	ascending colon	11	8.8
	transverse colon	3	2.4
	splenic flexure	3	2.4
	descending colon	7	5.6
	sigmoid colon	22	17.6
	rectosigmoid junction	28	22.4
	rectum	33	26.4
	All	*125*	*100.0*
Localization			
	left (cecum – transverse colon)	93	74.4
	right (splenic flexure – rectum)	32	25.6
	All	*125*	*100.0*
pT category			
	1	3	2.4
	2	12	9.6
	3	80	64.0
	4a	22	17.6
	4b	8	6.4
	All	*125*	*100.0*
pN category			
	0	59	47.2
	1a	14	11.2
	1b	15	12.0
	2a	12	9.6
	2b	25	20.0
	All	*125*	*100.0*
pM category			
	0	104	83.2
	1a	12	9.6
	1b	9	7.2
	All	*125*	*100.0*
